# Thermal Destabilization of Collagen Matrix Hierarchical Structure by Freeze/Thaw

**DOI:** 10.1371/journal.pone.0146660

**Published:** 2016-01-14

**Authors:** Altug Ozcelikkale, Bumsoo Han

**Affiliations:** 1 School of Mechanical Engineering, Purdue University, West Lafayette, Indiana, United States of America; 2 Weldon School of Biomedical Engineering, Purdue University, West Lafayette, Indiana, United States of America; 3 Birck Nanotechnology Center, Purdue University, West Lafayette, Indiana, United States of America; The Ohio State University, UNITED STATES

## Abstract

This study aims to characterize and understand the effects of freezing on collagen structures and functionality. Specifically, thermodynamic destabilization of collagen at molecular- and fibril-levels by combination of low temperatures and freezing were experimentally characterized using modulated differential scanning calorimetry. In order to delineate the effects of sub-zero temperature and water-ice phase change, we hypothesized that the extent of destabilization can be determined based on post-thaw heat induced thermal denaturation of collagen. It is found that thermal denaturation temperature of collagen in hydrogel decreases by 1.4–1.6°C after freeze/thaw while no such decrease is observed in the case of molecular solution. The destabilization is predominantly due to ice formation. Exposure to low temperatures in the absence of ice has only minimal effect. Calorimetry measurements combined with morphological examination of collagen matrices by scanning electron microscopy suggest that freezing results in destabilization of collagen fibrils due to expansion of intrafibrillar space by ice formation. This fibril-level damage can be alleviated by use of cryoprotectant DMSO at concentrations as low as 0.5 M. A theoretical model explaining the change in collagen post-thaw thermal stability by freezing-induced fibril expansion is also proposed.

## Introduction

Collagen is the primary structural component of tissue extracellular matrix (ECM) and plays a critical role for tissue mechanical integrity and functionality [1, 2]. Collagen ECM is a porous mesh of collagen fibrils, saturated by an interstitial fluid where individual fibrils are formed by self-assembly of tropocollagen molecules [3]. This hierarchical structure of collagen regulates various cell-cell and cell-matrix interactions [4–6]. Thus, collagen’s stability is critical to the functionality of native and engineered tissues. Due to significance of burn care and hyperthermic therapies [7], changes in collagen-based biomaterials by elevated temperatures have been well recognized [8]. However, thermal treatments where native/engineered biomaterials are exposed to a combination of sub-zero temperatures and freezing conditions have drawn less attention so far [9]. It is anticipated that these low temperature treatments can also alter the structure of collagen at multiple levels of its hierarchy as well as its post-thaw properties and functionality. Understanding the mechanisms by which sub-zero temperatures and freeze/thaw (F/T) alter collagen is crucial for development of successful cryopreservation [10, 11] and cryosurgery [12] technologies that utilize low temperature treatments to achieve long-term preservation of tissues and organs or selective destruction of tissues.

Effects of F/T on bulk tissue properties and microstructure of collagenous biomaterials have been extensively studied. There are numerous studies showing changes in thermal and mechanical properties of collagenous tissues upon F/T [13–15]. Changes in matrix-level properties such as porosity and hydration are well explained by local deformations that arise from dynamic mechanical interactions of ice with surrounding collagen matrix and interstitial fluid [14, 16, 17]. In contrast, effects of low temperature treatments on collagen’s lower hierarchies remain unexplored. This constitutes a critical gap in our understanding of freezing damage since bulk thermal and mechanical properties of collagenous tissues are greatly influenced by collagen’s molecular and fibril-level organizations [18–20]. For instance, tendons show decreased thermal stability and denaturation enthalpy when collagen fibrils become partially denatured by applied stress [21]. Bulk fracture behavior of collagen hydrogels are also determined by local fibril organization [22]. Therefore, our intention of this study is to explore the mechanisms of freezing damage sustained by collagen at fibril and molecular levels.

Thermodynamic destabilization of collagen by low temperatures and mechanical insult by ice formation are two alternative mechanisms that could explain previously reported F/T induced changes such as increase in fibril dimensions [13, 16] and disruption of fibril packing [23, 24]. Destabilization of proteins by freezing has been known for several decades and is considered to be due to combined effect of low temperatures, phase change induced dehydration, and freeze concentration of ionic constituents [2, 25, 26]. Thermodynamic origin of protein destabilization at low temperatures is explained by cold denaturation that refers to unfolding of protein native tertiary structure due to diminishing hydrophobic effect at those temperatures [27, 28]. Cold denaturation has been demonstrated for various proteins at above freezing temperatures, often in the presence of destabilizing agents [29–31], at sub-zero temperatures for proteins isolated by nano-confinement [32, 33] and also for cellular proteins under cryogenic storage conditions [34]. However, whether collagen undergoes cold denaturation upon freezing at a physiologically relevant tissue environment is currently unknown. If it does, cold denaturation may be responsible for the observed changes in fibril dimensions and architecture upon F/T. However, ice formation in intrafibrillar space can also result in deformations in collagen fibrils due to phase change induced expansion. These effects of temperature and ice formation need to be delineated to understand the origins of freezing damage in collagen’s lower hierarchies.

Although protein cold denaturation has been previously investigated by differential scanning calorimetry (DSC) [35], nuclear magnetic resonance [29, 30, 32] and Fourier transform infrared spectroscopy [34], direct measurement of collagen cold denaturation by these techniques in a physiological environment remains to be challenging due to interference by thermal and optical signatures associated with freezing. Therefore, in this study, we consider post-thaw measurement of heat-induced thermal denaturation of collagen as an alternative way to identify residual effects of cold denaturation. A similar approach was previously adopted to study cold denaturation of cellular proteins [34]. Thermal denaturation of collagen conveniently occurs above the phase change temperature and its kinetics are sensitive to molecular packing and hydration of collagen fibrils [18, 36]. As a result, changes in post-thaw thermal denaturation characteristics can be indicative of fibril-level structural changes that occur upon F/T. Despite its potential uses, there have only been a few studies of post-thaw denaturation in collagenous tissues. Among those, a net decrease in denaturation temperature was observed in tendons after storage at -80°C [13]. In contrast, thermal stability was enhanced in frozen and thawed (F/T) arteries [14]. These tissue dependent results indicate a need for systematic characterization of post-thaw thermal stability of collagen.

In this study, we address the above gaps by providing measurements of post-thaw thermal stability of collagen at multiple levels of its hierarchy using modulated temperature differential scanning calorimetry (MTDSC). Measurements were performed on collagen molecular solutions as well as on hydrogels with different collagen concentrations. Since hydrogels exhibit a fibrous mesh microstructure that is absent in molecular solution, it was possible to relate the F/T induced thermal stability changes of collagen to its distinct structures at matrix, fibril and molecular levels. In addition, morphological examination of collagen fibrils and network was performed with scanning electron microscopy to assess alterations in collagen matrices by F/T. The results were discussed to delineate the effects of ice formation and thermodynamic effects of low temperatures. The results were further analyzed by a computational model developed to relate F/T induced changes in collagen’s post-thaw thermal stability to fibril deformations.

## Materials and Methods

### Collagen Molecular Solution and Hydrogels

Rat tail collagen type-1 (Corning, Inc. NY) was purchased from the manufacturer as a concentrated stock solution. In order to study collagen in macromolecular form, the stock solution was diluted to a final collagen concentration of 3 mg/mL in 20 mM acetic acid. The resulting molecular solution of collagen remained unpolymerized when kept at room temperature for the duration of experiments. In addition, hydrogels exhibiting fibril and matrix-level structures of collagen were prepared according to manufacturer’s protocol. Appropriate amounts of 10X Dulbecco's Phosphate-Buffered Saline (10X DPBS, Life Technologies), 1.0 N NaOH, cell culture grade distilled water, and collagen stock solution were mixed together in a centrifuge tube on ice in the given order, resulting in a solution with neutral pH and isotonic ionic strength. This neutralized collagen solution had a final collagen concentration of either 3 or 6 mg/mL. Prepared solution was then dispensed into a chamber slide (Nunc Lab-Tek II, Thermo Scientific) and allowed to polymerize for 1 hour at 37°C in a CO_2_ incubator. Afterwards, isotonic saline solution (1X DPBS) was added on top of the hydrogel to prevent dehydration and incubation was continued until the experiments next day.

### Measurement of Collagen Thermal Denaturation by MTDSC

Circular hydrogel specimens were isolated by a 3 mm biopsy punch and placed in DSC pans. For molecular solution experiments, 10μL of solution was directly dispensed in the DSC pan. Pans were hermetically sealed to prevent dehydration during the experiments and were loaded in a differential scanning calorimeter (Q200, TA Instruments, IL) which was previously calibrated with indium, water and sapphire. Measurements were taken during heating from 25 to 60°C (molecular solution) or 30 to 65°C (hydrogel) with an underlying heating rate of 1°C/min. A sinusoidal temperature modulation with amplitude of 0.75°C and period of 80 seconds was superimposed with the underlying linear temperature program. Use of temperature modulation enabled deconvolution of specific heat signal into reversing and non-reversing components [37]. Heat absorption associated with thermal denaturation reaction enthalpy was observed exclusively in the non-reversing signal as an endothermic peak consistent with previous studies [38–40]. Change in the sensible specific heat during denaturation on the other hand resulted in a subtle shift in reversing specific heat signal (S1 Fig). Denaturation metrics were quantified based on the non-reversing specific heat after a linear baseline subtraction. Denaturation temperature, *T*_*d*_, was determined as the temperature attained at peak maximum. Temperature span of the transition was reported as peak width at half height, Δ*T*_1/2_, and denaturation enthalpy, Δ*H*_*d*_ was calculated as the area under the peak. Measurements were performed 3–4 times for each treatment group and results were averaged based on standard statistical methods.

### Freeze/Thaw Treatments

For freezing temperature studies, samples were cooled to either -20°C or -60°C in the DSC cell at a rate of 50°C/min, kept isothermal for 2 minutes, and warmed back to 20°C with 50°C/min. MTDSC measurements were initiated within 10 minutes of thawing.

In order to investigate the effects of ice formation, sample prepared in a DSC pan was placed on a temperature controlled stage (MDS 600, Linkam, U.K.) and monitored for ice nucleation while stage temperature was decreased to -15°C with a rate of 10°C/min and maintained at that temperature for 2 minutes. Small volume of the DSC sample enabled substantial supercooling allowing it to remain ice-free unless ice nucleation was deliberately induced by a liquid nitrogen cooled needle. Ice formation controlled this way enabled preparation of samples that were either supercooled and ice-nucleated, or supercooled only.

Different ice formation kinetics were obtained with a procedure similar to above. Samples were first cooled to -2°C where ice was nucleated. The presence of small ice crystals in the sample was verified under a microscope. Then, the samples were cooled to -20°C with two different cooling rates at 1 or 50°C/min. These two cooling rates were observed to generate different rates of ice growth in the samples.

For scanning electron microscopy, collagen samples prepared in chamber slides were placed on a directional solidification stage [17] and frozen by a temperature gradient developed from -60°C to 4°C across a 6 mm gap. During this process only half of the sample was frozen while the rest remained unfrozen.

For experiments with cryoprotectant dimethyl sulfoxide (ME_2_SO, DMSO), hydrogels were incubated in saline solution including DMSO at concentrations ranging between 0.25 M and 1 M for 30 minutes before freezing. After 10 minutes of passive thawing at room temperature, samples were sectioned and transferred to DSC pans followed by freezing in the DSC cell as explained before. Since DMSO in the hydrogels was not unloaded after F/T, the samples prepared in this way contained DMSO during the MTDSC measurements. In order to control for possible confounding effects of DMSO in the measurements, experiments were also performed on DMSO loaded unfrozen collagen hydrogels and the results were compared with 0 M DMSO case.

To explore the relative effects of freezing-induced mechanical expansion and freeze-concentration of solutes, collagen hydrogels were treated with a hypertonic solution (10X concentrated saline) for 20 min followed by repeated washing in isotonic saline for 30 min. This procedure approximated the transient concentration increase of solutes during F/T. Parts of hydrogels treated with hypertonic solution were then F/T to -60°C and post-thaw denaturation was measured as before.

### Scanning Electron Microscopy Imaging

Hydrogels were F/T on a directional solidification stage as described before and passively thawed at room temperature for 15 minutes. The samples were then immediately fixed with 2.5% glutaraldehyde and 2% formaldehyde in 1X DPBS, and stored in fixation solution at +4°C overnight. Afterwards circular sections were punched out from unfrozen and F/T regions, dehydrated against ethanol and dried in a CO_2_ critical point dryer. Through the sample preparation, the sizes of specimens unfrozen or F/T with DMSO remained unchanged while notable shrinkage was observed for the specimens frozen and thawed without DMSO. Specimens were then imaged using a scanning electron microscope (Nova NanoSEM 450, FEI, OR). At least 3 randomly selected fields of views were imaged per sample. The fibril diameters were estimated by manually tracing the boundaries of approximately 100 fibrils per each group by ImageJ [41].

### Kinetic Model of Collagen Denaturation

In this study, we considered collagen thermal denaturation as a single step irreversible rate reaction based on earlier work of Miles et al. [38]. The denaturation reaction is,

$$N_{UF}\overset{k_{UF}}{\to }D_{UF}\text{ and }N_{F/T}\overset{k_{F/T}}{\to }D_{F/T}$$ (1)

Here *N* and *D* are the native and denatured states of collagen, respectively. Both unfrozen (UF) and frozen/thawed (F/T) collagen is assumed to go through the same reaction, but at possibly different rates.

For both UF and F/T, kinetics of denaturation is described by the rate law,

$$\dot{\alpha }=k(T)f(\alpha )$$ (2)

where *α* is the extent of conversion from native to denatured state, $\dot{\alpha }$ is the conversion rate, *k* is the rate constant and *f* is the reaction model. Arrhenius type temperature (*T*) dependence for the rate constant and an *n*^th^ order reaction model [42] were assumed as follows:

$$k(T)=A\text{ exp}\left(\frac{-E_{a}}{RT}\right),\;f(\alpha )={\left(1-\alpha \right)}^{n}$$ (3)

In Eq 3, *A* is commonly referred as the pre-exponential factor and *E*_*a*_ is the activation energy. These together with the reaction order *n*, are the kinetic parameters that are to be estimated by fitting the above model to experimental data by an optimization routine developed in MATLAB^®^.

### Theoretical Model of Fibril Expansion and Thermal Stability

**Fig 1:**
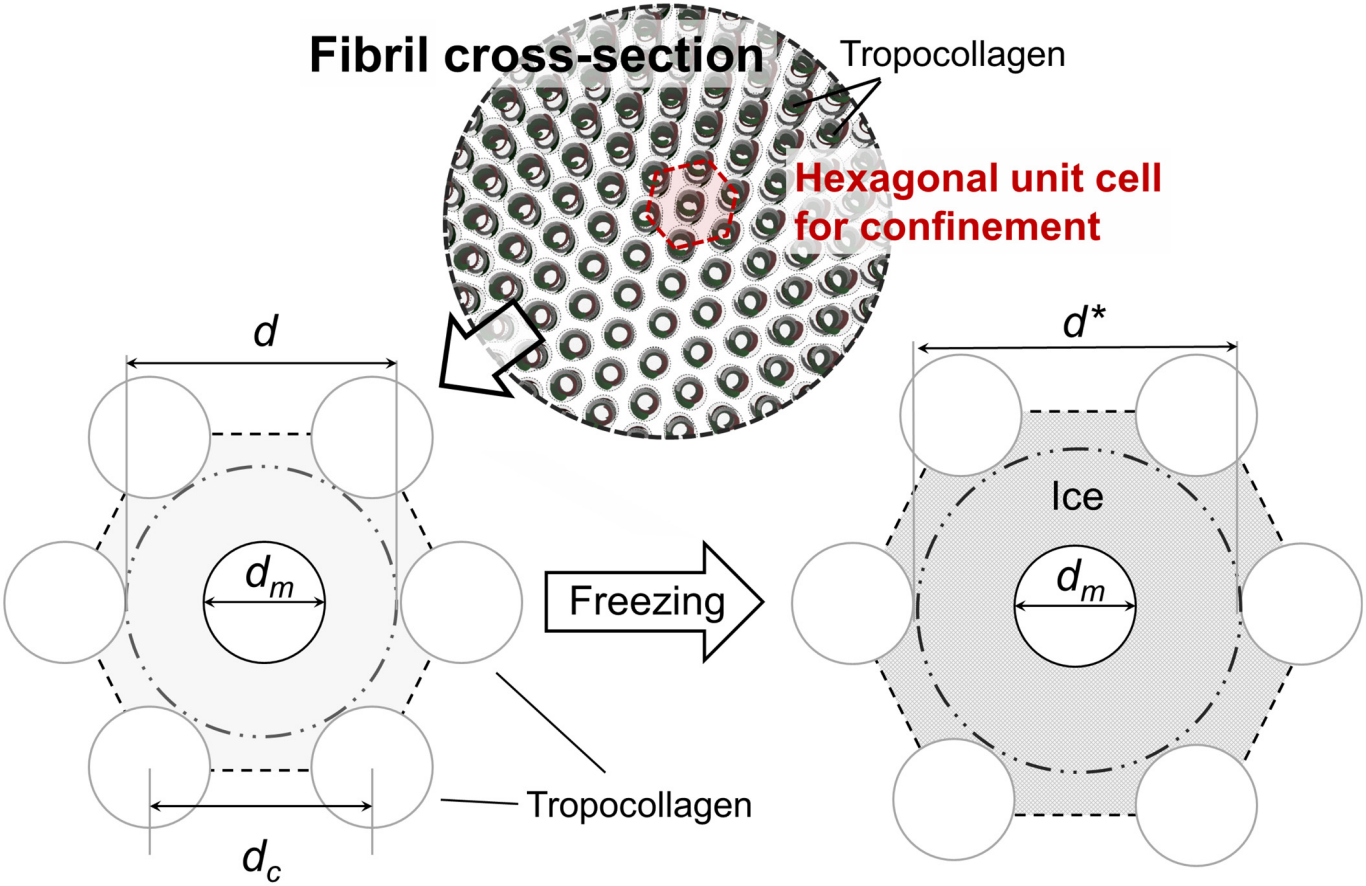
Freezing-induced expansion of collagen fibril. Average confinement of single collagen molecule in the fibril is modeled by considering an equilateral hexagonal unit cell for confinement based on a quasi-hexagonal packing state [44, 45]. Unit cell expands as intrafibrillar fluid freezes. *d*_*m*_ is tropocollagen nominal diameter, *d* is confinement diameter, *d*_*c*_ is the length of a side of the unit cell.

Freezing induced expansion of fibril was modeled by a unit cell / representative volume approach that was previously used in modeling freezing-induced tissue-level expansion [43]. Mean confinement of single collagen molecule by its neighbors in a fibril is calculated based on the dimensions of a unit cell assuming quasi-hexagonal packing of collagen fibrils [44, 45] as shown in Fig 1. The quasi-hexagonal packing is a simplification over the microfibrillar packing of collagen [46] by neglecting the local variations in intermolecular distances by molecular cross-links and longitudinal offset of tropocollagen within the fibril. This simplified model is considered adequate to evaluate the mean confinement effects induced on a tropocollagen molecule by its neighbors. The space between the molecules is filled with intrafibrillar fluid, which is considered to expand upon freezing and result in an increase in unit cell dimensions.

The increase of thermal stability of collagen upon assembly into fibrils is based on the reduction of configurational entropy due to molecular confinement effects [18, 47]. Therefore, the observed decrease in thermal stability upon freezing of fibrils is expected to be due to relaxation of the confinement. This entropic destabilization effect can be expressed by a relationship between activation entropy of denaturation Δ*S*^↕^, and confinement diameter, *d*, based on statistical mechanics modeling of a polymer in a tube [18, 48],

$$\Delta S^{↕}=\Delta S_{o}^{↕}-\frac{4\pi RNb^{2}}{d^{2}}$$ (4)

where $\Delta S_{o}^{↕}$ is the activation entropy of denaturation under unconfined state, such as dilute molecular solution. *N* is the number of links in the thermally labile domain of collagen (*N* = 65 [18]), *b* is the effective bond length that is taken to be the same as the actual bond length (*b* = 0.35 nm [18]) and R is the universal gas constant. In this derivation, it is assumed that the activated state of collagen acts as a Gaussian chain while the native triple helix is a rigid worm-like polymer. Therefore confinement effects become significant only for the activated state [49, 50].

The activation entropy is related to the pre-exponential factor, *A*, in the previously described kinetic model as:

$$A=B(T)\text{exp}\left(\frac{\Delta S^{↕}}{R}\right)$$ (5)

where B(T) is a slowly varying function of temperature [51].

By considering Eqs 4 and 5 for an arbitrary configuration with confinement diameter *d*_*2*_ in comparison to a reference configuration with confinement diameter *d*_*1*_, we obtain:

$$\frac{A_{2}}{A_{1}}=\text{exp}\left[4\pi Nb^{2}\left(\frac{1}{d_{1}}-\frac{1}{d_{2}}\right)\right]$$ (6)

Therefore, reaction kinetics of an arbitrary confinement level can be predicted based on the reaction kinetics and confinement level of the reference state.

The amount of this expansion mainly depends on fibril native i.e. unfrozen porosity. Upon expansion, confinement diameter, *d*, increases resulting in a decrease in molecular confinement. The relative increase in unit cell dimensions is given by:

$$\frac{d_{c}^{*}}{d_{c}}=\sqrt{1+\phi \left(\frac{\rho_{f}}{\rho_{i}}-1\right)}$$ (7)

where *d*_*c*_ and $d_{c}^{*}$ are the length of a side of the unit cell at unfrozen and frozen states respectively. *ϕ* is the fibril porosity at unfrozen state. *ρ*_*f*_ and *ρ*_*i*_ are the densities of intrafibrillar fluid and ice, respectively. Porosity is calculated from geometric relationships as:

$$\phi =1-\frac{\pi }{2\sqrt{3}}{\left(\frac{d_{m}}{d_{c}}\right)}^{2}$$ (8)

where *d*_*m*_ is the collagen molecular diameter that was varied between 1.3 and 1.5 nm [18, 52]. The confinement diameter is obtained as *d* = 2*d*_*c*_-*d*_*m*_, also based on unit cell geometry.

Eqs 6 and 7 were used together with kinetic model to predict the denaturation temperature decrease with freezing-induced fibril expansion.

### Statistical Analysis

Differences in treatment means were tested by one-way ANOVA. Multiple comparisons were performed by Tukey’s test. The differences were considered statistically significant when *p-value* was less than 0.05. Experimental data are reported in terms of mean ± standard deviation.

## Results

### Shift in Endothermic Peak of Denaturation by F/T

**Fig 2:**
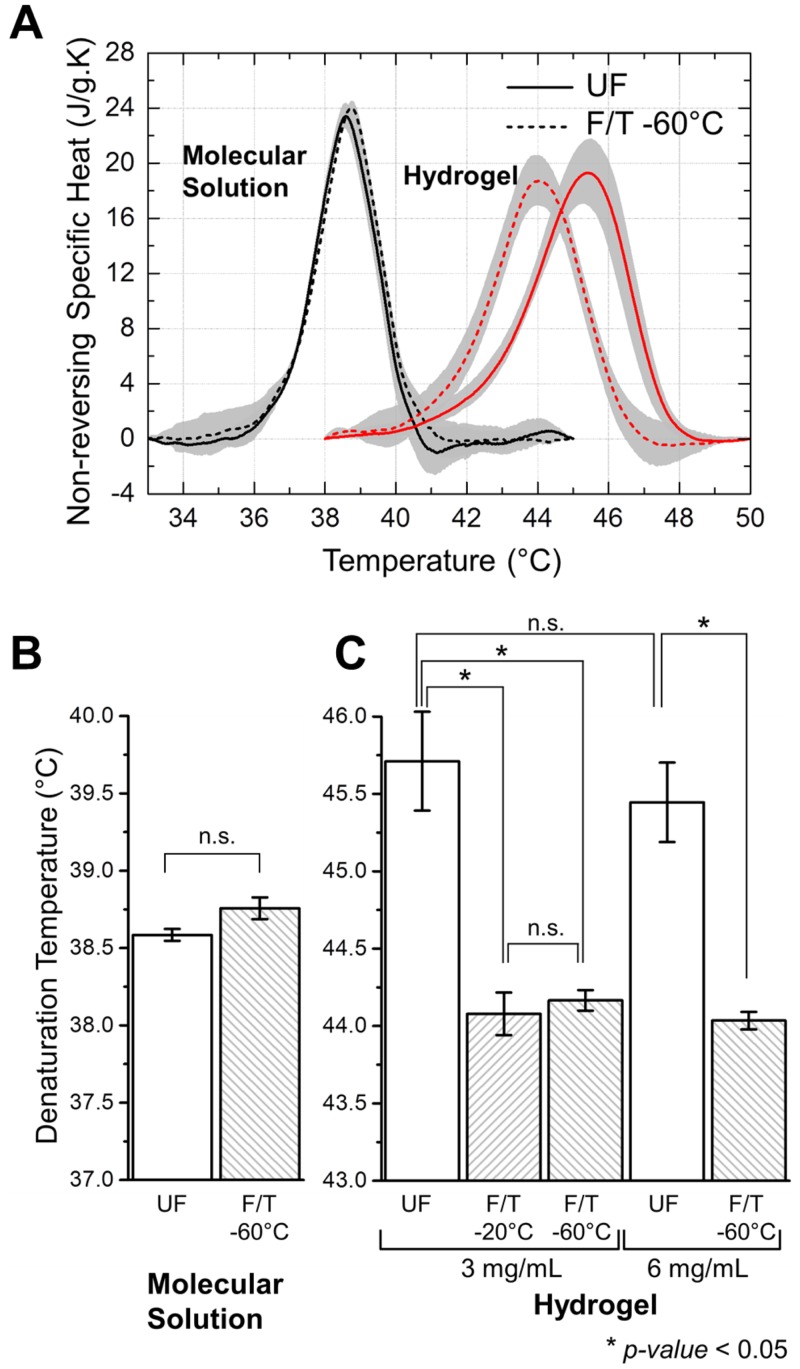
(A) Representative MTDSC heating thermograms that show endothermic peaks of denaturation for F/T versus UF collagen in hydrogels and molecular solutions. Lines and shaded regions indicate the mean and standard deviation respectively. Denaturation temperature of collagen in (B) molecular solution and (C) hydrogel. ‘*’ indicates significant difference (*p* < 0.05).

Heat absorbed by collagen during thermal denaturation is observed as an endothermic peak in a DSC thermogram [39]. Representative MTDSC thermograms showing endothermic peaks of denaturation for F/T and UF collagen are presented in Fig 2A. It is found that F/T treatment has a destabilizing effect specific to collagen hydrogel. The peak of denaturation in F/T hydrogels occurred at lower temperatures compared to UF. However, a similar shift in temperature was not observed in the case of F/T molecular solution.

Fig 2B and 2C present the denaturation temperatures, *T*_*d*_, determined from the location of endothermic peak. Collagen as a macromolecule maintained its thermal stability even after F/T to -60°C (Fig 2B). Peak width and denaturation enthalpy of collagen in molecular solution were also similar for F/T and UF cases with differences not statistically significant (S1 Table). In the case of hydrogels on the other hand, F/T resulted in a significant decrease in *T*_*d*_, ranging from 1.4 and 1.6°C, regardless of the freezing temperature of either -20°C or -60°C. The amount of the decrease also did not depend on collagen density of the hydrogel (p > 0.05) (Fig 2C).

### Effects of Ice Formation on Collagen Post-Thaw Thermal Stability

**Fig 3:**
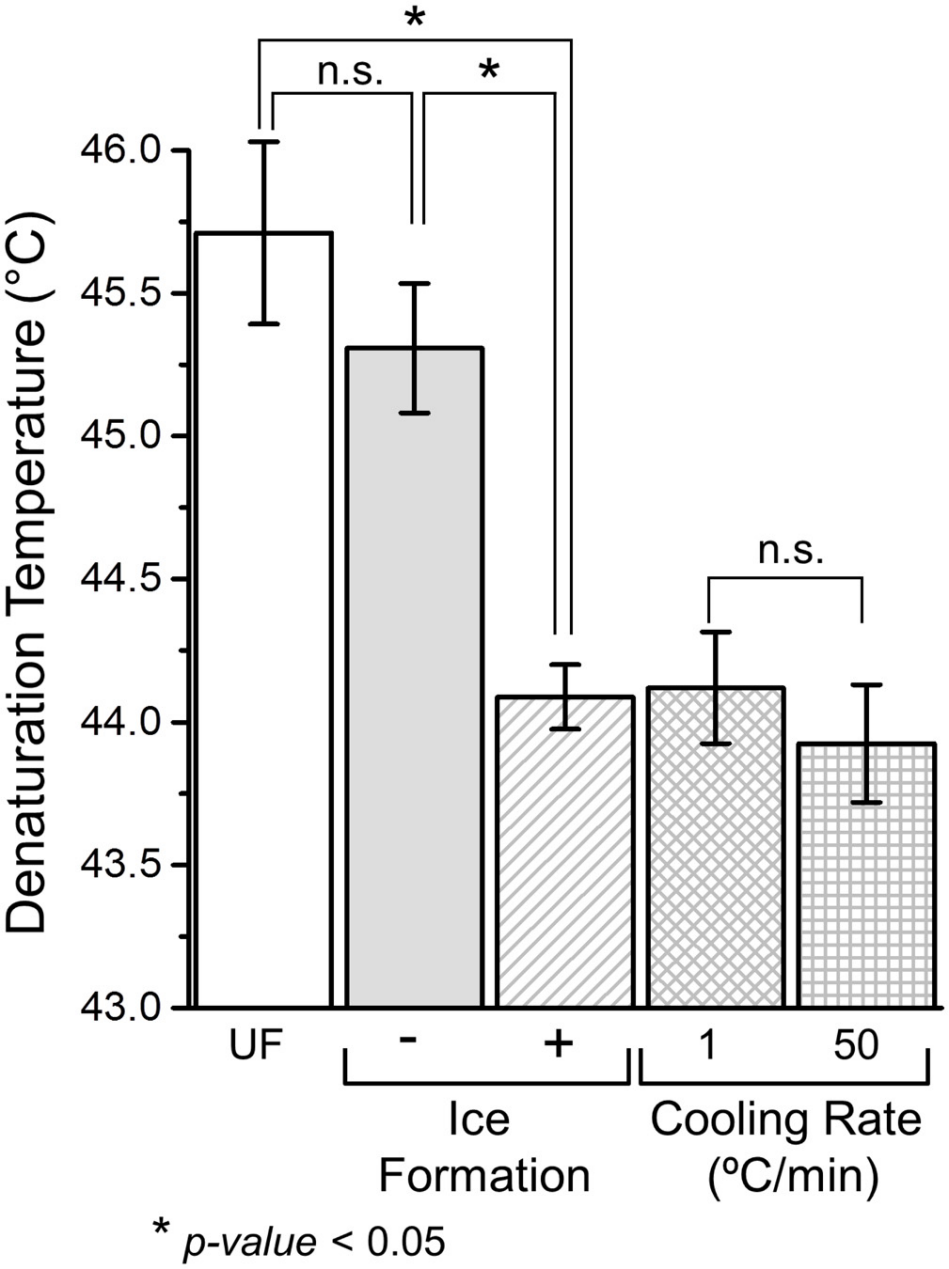
Effects of F/T conditions on post-thaw denaturation temperature. ‘*’ indicates significant difference (*p* < 0.05).

In order to distinguish between the mechanical effect of ice formation and thermodynamic effects of low temperatures, experiments were conducted on collagen hydrogels either ice nucleated or supercooled with the same temperature history. Consistent with the previous results, the hydrogels that experienced ice formation denatured at a significantly lower temperature than UF as shown in Fig 3. Furthermore, the *T*_*d*_ decrease caused by ice formation was comparable to those from freeze/thaw at -20°C and -60°C. Interestingly hydrogels supercooled with the same temperature history also had a lower *T*_*d*_ than UF. However, the difference was not statistically significant. Effects of cooling rate were also investigated by considering F/T treatments to -20°C with either 1 or 50°C/min. It was found that higher cooling rate resulted in a slightly lower *T*_*d*_ than lower cooling rate. However, the difference between the two cases was not statistically significant.

Since ice formation results in both mechanical expansion and increased concentration of solutes in collagen, we compared denaturation of collagen hydrogels before and after hypertonic solution treatment as well as F/T with and without hypertonic solution treatment. It was found that hypertonic treatment resulted in a net decrease in denaturation temperature that was significantly less then F/T alone (S3 Fig). When hypertonic solution and F/T were combined, the magnitude of the denaturation temperature decrease was in excess of either treatment.

### Preservation of Collagen Post-Thaw Thermal Stability by Cryoprotectant

**Fig 4:**
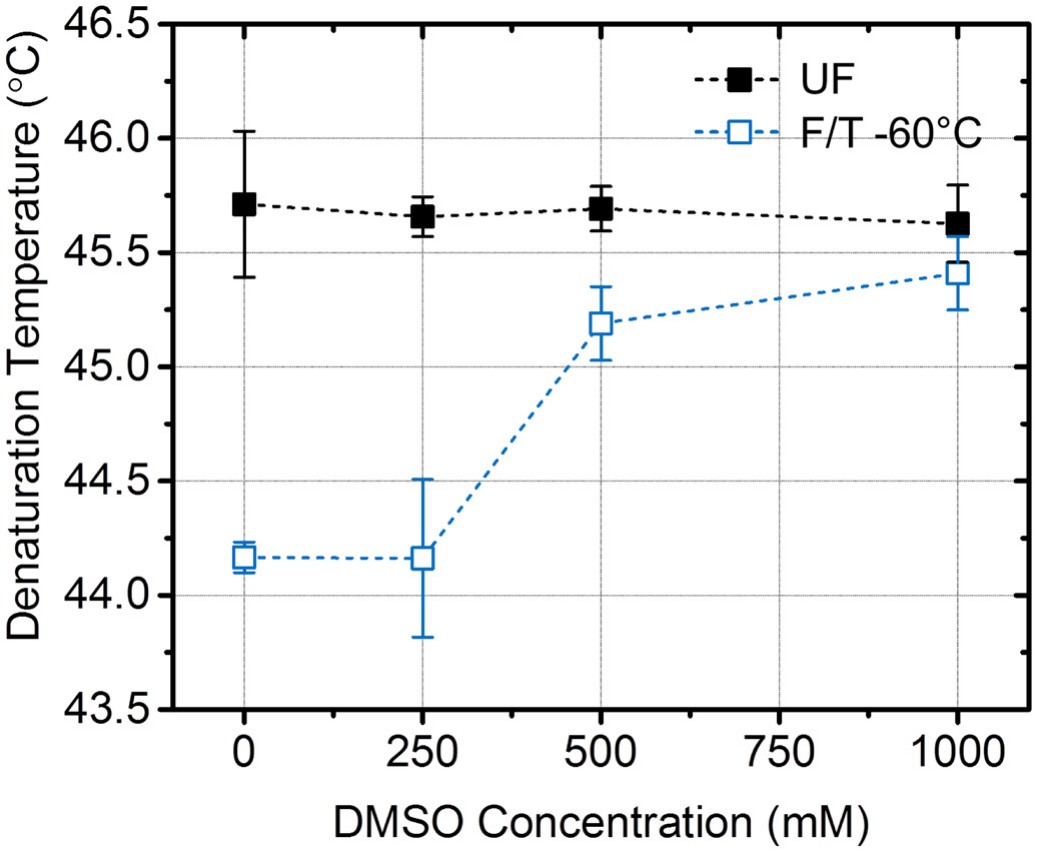
Recovery of collagen post-thaw thermal stability by use of cryoprotectant, DMSO.

During cryopreservation, cells and tissues are often loaded with cryoprotectants such as dimethyl sulfoxide (DMSO) that can help reduce freezing damage by decreasing the amount of phase-change induced expansion [53]. To test whether DMSO can preserve collagen post-thaw thermal stability, hydrogels were treated with up to 1 M DMSO in physiological buffer prior to F/T treatment. Possible effects of DMSO on MTDSC measurements were controlled for by treating UF control as well. It was observed that the thermal stability of F/T hydrogels were gradually recovered to unfrozen levels with increasing concentration of DMSO as shown in Fig 4. On the other hand, DMSO didn’t change the denaturation temperature of UF. Concentrations above 0.25 M DMSO effectively increased the denaturation temperature and 1 M DMSO was sufficient for near complete recovery of thermal stability.

**Fig 5:**
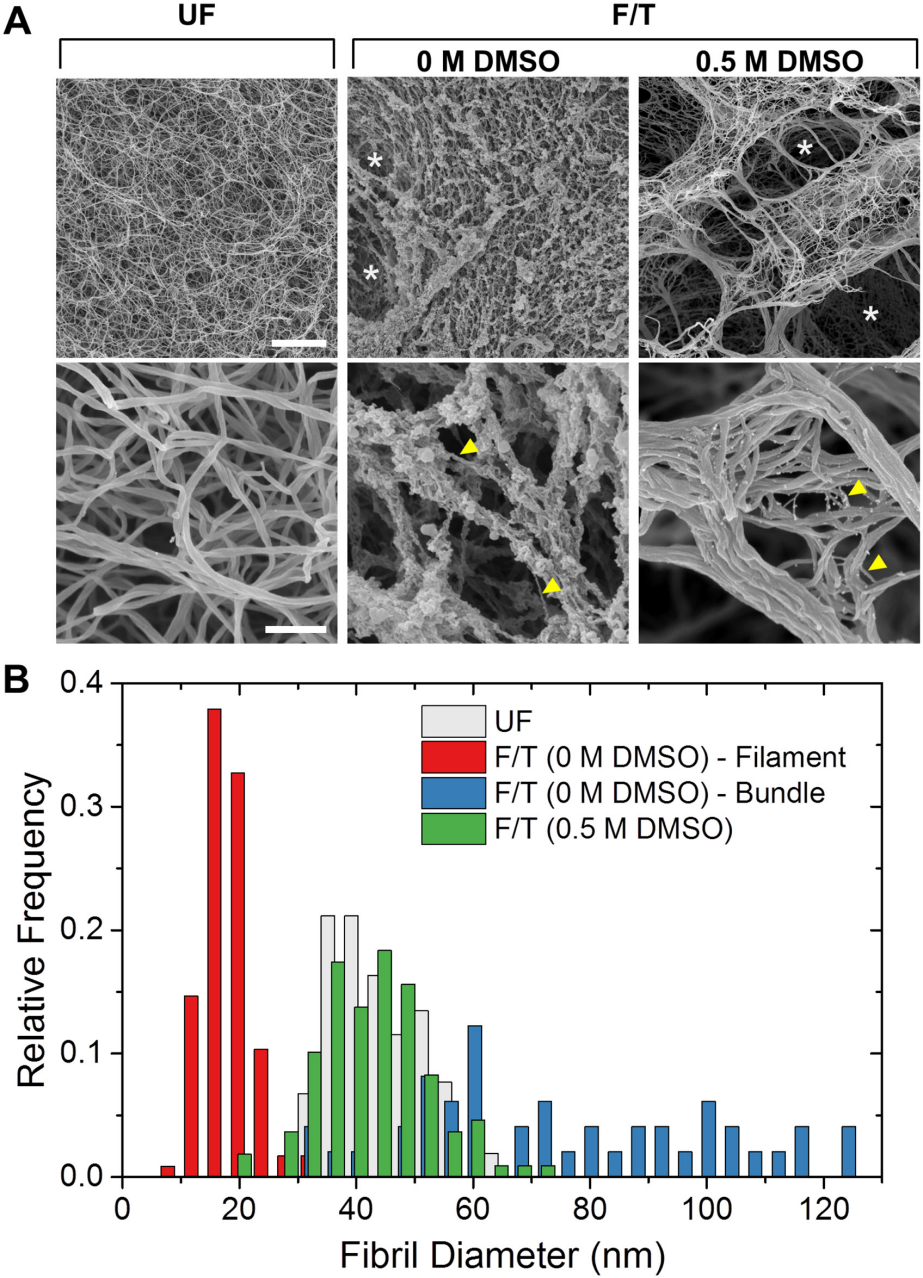
Freezing-induced morphological changes in collagen hydrogels at network and fibril levels. (A) Representative SEM images. Scale bars are 5 μm and 500 nm for top and bottom panels respectively. (B) Hydrogel fibril diameter distributions.

Examination of collagen hydrogel microstructure with scanning electron microscopy revealed dramatic changes in both network and fiber level upon F/T as shown in Fig 5A. Hydrogels frozen with and without DMSO both showed large pores (white arrows in top panel) which were not present in unfrozen controls. The sizes of these pores were not comparable due to differential shrinkage of SEM specimens during preparation. The case of 0.5 M DMSO showed fibrils bundled together. F/T in the absence of DMSO also disrupted the fibril morphology. The case of 0 M DMSO showed collection of thin filaments with a mean diameter of *d* = 18.1 ± 4.1 nm (yellow arrows, bottom middle). These filaments seemed to be a part of multiple, thick, but loosely packed bundles (d = 83.9 ± 36.0 nm) which could have originated from fibrils that have unraveled during F/T due to expansion. This was in contrast to the smooth, uniformly sized fibrils observed in unfrozen control (*d* = 43.3 ± 7.4 nm). The extent of this fibril level damage was alleviated by use of DMSO during freezing. The case of 0.5 M DMSO also showed mostly intact fibrils with diameters, *d* = 42.9 ± 9.4 nm, similar to that of unfrozen control. The distributions of fibril diameters are shown in Fig 5B.

### Modeling of Thermal Destabilization by Freezing-Induced Fibril Expansion

**Fig 6:**
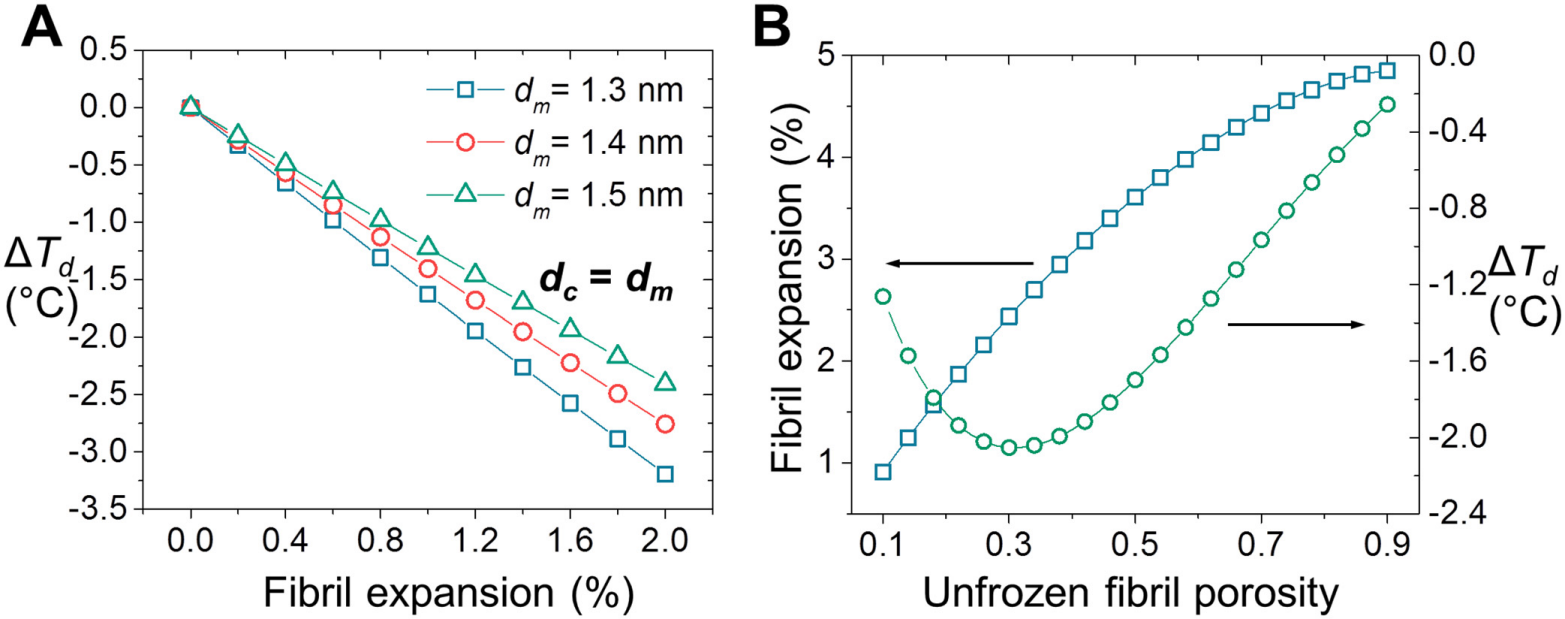
Prediction of denaturation temperature decrease upon F/T by computational modeling. (A) The change in denaturation temperature upon hypothetical expansion of a tightly packed fibril (minimum porosity). (B) The amount of freezing-induced fibril expansion and change in denaturation temperature as a function of unfrozen fibril porosity.

A computational model was developed to explain the observed change in *T*_*d*_ by F/T by considering the freezing-induced expansion of collagen fibrils and associated relaxation of collagen molecular confinement in fibrils. First, Arrhenius parameters of denaturation were determined by kinetic analysis of denaturation endothermic peaks (S2 Fig and S2 Table). Then, denaturation thermograms were simulated with varying reaction kinetics based on Polymer-in-a-box Theory [48] and F/T induced changes in confinement as illustrated in Fig 1. Fig 6A shows the change in *T*_*d*_ as a function of fibril expansion in the case of a tightly packed fibril. It is seen that even a small amount of increase in fibril volume, less than 2%, results in significant decrease of *T*_*d*_. The effect is almost linear and shows small variation with respect to reported values of collagen molecular diameter [18, 52]. Fig 6B shows the effect of unfrozen fibril porosity on freezing-induced expansion of the fibril and the associated decrease in *T*_*d*_. Overall, the magnitude of the decrease was in agreement with the experiments. In addition, fibril porosity is predicted to be an important factor that can affects the extent of F/T induced decrease in *T*_*d*_. The decrease in *T*_*d*_ shows a biphasic behavior with respect to fibril porosity with the greatest amount of decrease occurring at an intermediate porosity level. The experimentally observed range of Δ*T*_*d*_ i.e. from -1.4 to -1.6°C, corresponds to values of fibril porosity in the ranges of 0.12–0.15 and 0.5–0.6. The latter range is in agreement with previous estimates of porosity based on changes in fibril dimensions upon drying/rehydration [54].

## Discussion

### Freezing-Induced Destabilization of Collagen as a Fibril-Level Phenomenon

Our results indicate that collagen matrix hierarchical structure is destabilized upon F/T as observed through a decrease in post-thaw thermal denaturation temperature. This decrease is specific to hydrogels and is not observed in molecular solution (Fig 2A and 2B). Therefore, even if there are any changes that take place during F/T at collagen molecular level, these changes are not lasting and therefore not reflected in the post-thaw thermal stability. In addition, results presented in Fig 2C indicate the extent of this F/T effect is not affected by changes in matrix structure and properties induced by varying collagen concentration in hydrogel e.g. porosity, fibril density and connectivity. These results together suggest that the observed destabilization due to F/T is caused by changes in fibril-level structure.

### Freezing-Induced Fibril Expansion

We next investigated the mechanisms of the thermal destabilization of collagen and identified that it is ice formation, rather than exposure to low temperatures, that mainly caused the observed decrease in denaturation temperature (Fig 3). For the temperature regime studied (-15°C to -60°C), purely thermodynamic effects of low temperatures were indeed found to be secondary when compared to effects of ice formation. Our experiments indicate that F/T induced denaturation temperature decrease is abrupt upon ice formation and is also not significantly affected by the cooling rates. The amount of denaturation temperature change was similar for cooling rates between 1 and 50°C/min. As a result, cooling rate does not seem to be a major factor in determining the freezing-induced changes in collagen fibrils within the range studied.

The present results imply that the freezing-induced destabilization of collagen is thought as a fibril-level biophysical process, specifically freezing-induced expansion of hydrated collagen fibrils. The expansion of intrafibrillar fluid upon freezing can cause mechanical stresses that eventually result in disruption of fibril packing and expansion of the fibril, which lowers the heat denaturation temperature of collagen. Since the heat denaturation temperature of collagen molecular solution did not change, this also supports that the destabilization is fibril-specific process. Use of cryoprotective DMSO lessened the change of heat denaturation temperature, which correlates with preserved fibril structure (Figs 4 and 5). Both results can be explained by the decrease in phase change induced expansion of intrafibrillar fluid associated with the addition of DMSO. The fibril expansion also could explain the F/T induced changes in fibril shape and dimensions reported in previous studies [13, 16, 23, 24].

In addition to physical expansion of intrafibrillar fluid, since freezing also elevate electrolyte concentrations, the collagen fibrils may also be destabilized by exposure to hypertonic concentration. In order to confirm this, hydrogels were exposed to a hypertonic saline with or without F/T, and corresponding heat denaturation temperatures were measured. The results are provided in S3 Fig. The results suggest that elevated salt concentration also destabilized the collagen fibrils but the extent is less. The protective effect of DMSO may also be interpreted by reduced electrolyte concentrations during freezing since DMSO not only decreases the amount of freezable water but also dilutes the freezing solution. Therefore, both freezing-induced fibril expansion and freeze concentration remain as possible alternative explanations for the observed decrease in collagen thermal stability. Further research is warranted to delineate or understand the interactions of these two mechanisms.

When considering the possibility of intrafibrillar ice formation it is also important to take into account the state of water confined in collagen fibril. When water is confined in small-scale pores, its freezing point is depressed due to geometric constraints on ice curvature associated with perturbation of its hydrogen bond network [55]. This phenomenon is thermodynamically described by the Gibbs Thomson equation that relates the freezing point depression of a liquid to the reciprocal of pore diameter [56]. The effect is most pronounced in pores with diameters on the order of a nanometer. For example, water freezes at -40 and -60°C when isolated in silica gels with cylindrical pores of 4.4 and 3 nm diameters, respectively [55, 57]. Mean lateral intermolecular distance in a fibril is in a similar range and is estimated to be within 1 to 5 nm depending on the porosity. Assuming a uniform collagen fibril, these indicate a confinement effect that could be significant enough to prevent formation of intrafibrillar ice under the F/T conditions studied.

However, there are inherent heterogeneities in collagen fibril structure that could allow formation of ice at specific sites. There is evidence that collagen fibrils have a tubular structure with a dense shell and a hollow core indicating presence of relatively large pores in the interior of the fibril. [58, 59]. In addition, there are variations in the amount of water along the longitudinal packing of fibril with greater amount of water being present in the gap region compared to overlap region [60]. The regions with pores that are larger than average could allow ice formation without significant super-cooling. This could also lead to a cascade effect where local freezing-induced expansion disrupts the lateral packing and enables propagation of ice into the other parts of the fibril. Therefore, formation of intrafibrillar ice is possible even when part of water in fibril is significantly confined. Future studies are granted to investigate the specific mechanisms of ice propagation within collagen fibrils.

### Relaxation of Collagen Molecular Confinement in Fibril by F/T

Freezing-induced fibril expansion is proposed as the mechanism of the observed destabilization of collagen, and relationship between the two is properly explained by theories of protein folding under confinement [47] such as Polymer-in-a-box model [48]. In particular, it has been previously shown that changes in confinement of collagen in fibrils by dehydration or additional cross-linkers change its thermal denaturation kinetics [18, 36]. Based on these, decreasing confinement is expected to destabilize native collagen by decreasing the entropic cost of unfolding. Therefore, relaxation of collagen molecular confinement in fibril by its freezing-induced expansion results in a decrease in thermal stability that is consistent with the experiments. This is demonstrated by a computational model in this study, where fibril porosity is found to be a significant factor in the degree of destabilization.

Results of our model are in agreement with the denaturation temperature decrease observed in tendons upon F/T [13]. However, an increase in denaturation temperature upon F/T is also reported where the increase is attributed to dehydration of the fibrils upon F/T [14]. The suggested mechanism for this change is the molecular confinement effect [18] that was also considered in our model. While our model does not explain the dehydration of fibrils upon F/T, the associated denaturation temperature increase can be simulated given information on extent of fibril dehydration. In this study, we focused on the fibril expansion hypothesis based on our observations of collagen hydrogels with DSC. The reasons behind tissue-dependent expansion or compaction of collagen fibrils will require further investigation that is beyond the scope of this study.

It is important to note that the model simulations presented here represent average effects since they do not take into account the variations in molecular packing due to inherent heterogeneities in collagen fibril at gap region and fibril core [58–60]. The presence of a hydration shell around the tropocollagen molecules is also not considered in the model. This is a 2 to 3 molecules thick layer that is composed of strongly bound water which remains unfrozen and does not contribute to freezing-induced expansion [55]. The presence of the hydration shell is expected to have a minor effect on the amount of freezing-induced fibril expansion.

### Implications to Cold Denaturation of Collagen by F/T

Our results indicate that thermal stability of collagen is preserved upon F/T when collagen is maintained at macromolecular state. In other words, short-term exposure to temperatures as low as -60°C does not have a lasting effect on molecular collagen. Irreversible denaturation of collagen during F/T under the studied conditions is unlikely since denaturation enthalpies were similar for cases with and without F/T treatment (S1 Table). If F/T caused collagen to denature irreversibly, one would expect to see a decrease in the enthalpy of post-thaw denaturation due to decreased number of native molecules available for denaturation following F/T. While irreversible denaturation of cellular proteins was reported at low temperature storage conditions [34], cold denaturation is commonly regarded as a reversible process [25]. Due to the post-facto nature of post-thaw measurements, the results presented in this study do not explain whether collagen has undergone such a transition during the cooling process and reverted back to its original state after F/T.

## Conclusion

In this study, post-thaw thermal stability of collagen was investigated at matrix, fibril, and molecular levels. It is found that thermal stability of collagen can be different before and after freeze/thaw. However, the difference is observed only in a hydrogel environment where collagen has assembled into fibrils. Supported by structural evaluation of collagen and biophysical modeling, these results indicate to a specific type of fibril damage that occurs due to freezing-induced expansion of intrafibrillar fluid. This is a structural/mechanical deformation process that elicits itself in post-thaw thermal stability metrics. This fibril-level damage could be alleviated by cryoprotectant DMSO at concentrations as low as 0.5 M. Since the structure of collagen fibrils plays a major role in determining thermal properties and load carrying functionalities, we expect the findings of this study to be useful for understanding the freezing-induced changes in bulk properties of collagenous biomaterials during cryopreservation and cryotherapy. The results will be particularly useful for proper interpretation of calorimetric studies that often need to employ specimens that are previously stored at frozen state.

## Supporting Information

S1 Fig Representative MTDSC heating thermogram of collagen hydrogel. The endothermic peak associated with thermal denaturation of collagen is predominantly observed in non-reversing specific heat signal.
(PDF)

S2 Fig Simulation of collagen denaturation based on experimentally determined reaction kinetics. (PDF)

S3 Fig Denaturation temperature change with hypertonic solution treatment, F/T and combination of the two treatments. (PDF)

S1 Table Collagen thermal denaturation peak characteristics for selected treatments. (PDF)

S2 Table Kinetic model parameters. The best fitting values of model parameters, reaction order, *n*, activation energy, *E*_*a*_, and the pre-exponential factor, *A*, are reported for selected F/T and UF cases.
(PDF)
