# Supplementary material for: Thermal Destabilization of Collagen Matrix Hierarchical Structure by Freeze/Thaw
Source: PLoS One. 2016 Jan 14;11(1):e0146660. doi: 10.1371/journal.pone.0146660 (PMC4713088; doi:10.1371/journal.pone.0146660)
Supplement: S1 Fig — The endothermic peak associated with thermal denaturation of collagen is predominantly observed in non-reversing specific heat signal. (PDF) [file pone.0146660.s001.pdf]

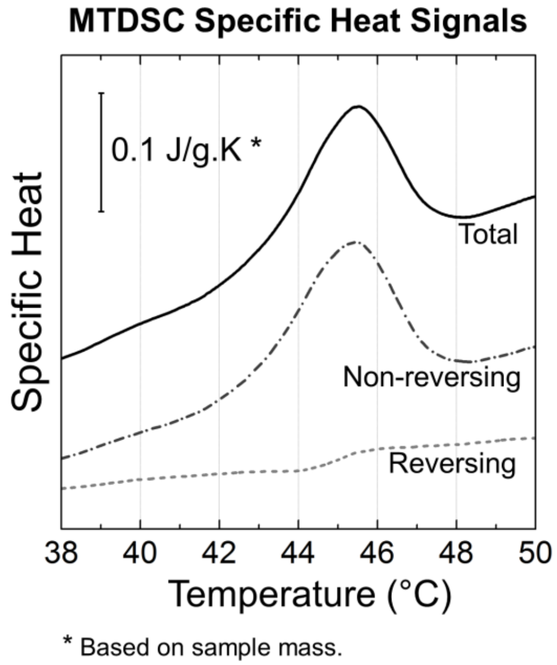

Fig S1. Representative MTDSC heating thermogram of collagen hydrogel. The endothermic peak associated with thermal denaturation of collagen is predominantly observed in non-reversing specific heat signal.

Fig S1 shows the three specific heat signals obtained from a representative MTDSC thermogram for collagen hydrogel. The total signal is the summation of reversing and non-reversing components and is equivalent to the signal that would be obtained by standard DSC. Collagen heat denaturation appeared as a distinct endothermic peak in non-reversing specific heat and was associated with only a subtle increase in reversing specific heat. The change in non-reversing specific heat is attributed to heat absorption associated with the denaturation reaction and is similar to what has been reported with conventional DSC [1]. The relatively small change in reversing specific heat is attributed to the sensible heat transfer to the sample and indicates an increase in partial specific heat upon denaturation of fibrillar collagen. The distinct peak in non-reversing signal provides more information on denaturation and is associated with higher signal-to-noise ratio than the reversing signal. Therefore, quantification of denaturation metrics was done using the non-reversing signal.

References

1. Miles CA, Burjanadze TV, Bailey AJ. The kinetics of the thermal denaturation of collagen in unrestrained rat tail tendon determined by differential scanning calorimetry. J Mol Biol. 1995;245:437–46.
