# Supplementary material for: Thermal Destabilization of Collagen Matrix Hierarchical Structure by Freeze/Thaw
Source: PLoS One. 2016 Jan 14;11(1):e0146660. doi: 10.1371/journal.pone.0146660 (PMC4713088; doi:10.1371/journal.pone.0146660)
Supplement: S2 Fig — (PDF) [file pone.0146660.s002.pdf]

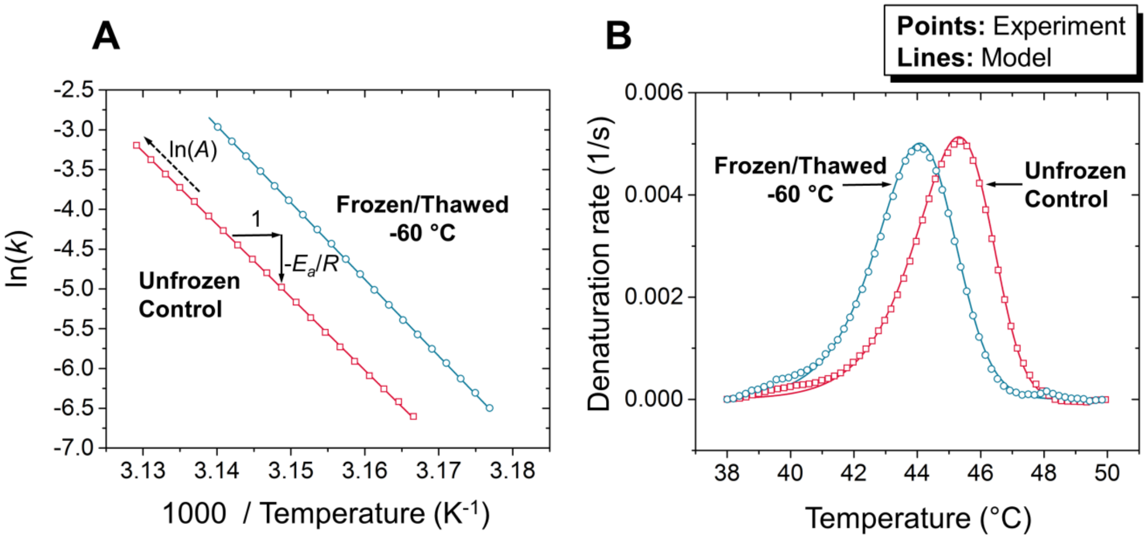

Fig S2. Simulation of collagen denaturation based on experimentally determined reaction kinetics. (A) Estimation of kinetic model parameters. (B) Comparison of simulated and experimentally obtained denaturation peaks. Experimental data is rarefied for illustration. Each plotted point corresponds to 100 data points.

Kinetic parameters necessary to simulate thermal denaturation were determined by fitting an irreversible rate law to experimental thermograms. The Arrhenius coefficients and reaction order were determined from the Arrhenius plot as illustrated in **Fig S2 A.** Thermograms that were simulated using these set of parameters were in excellent agreement with ones experimentally determined ($R^2 > 0.996$ for hydrogels and $R^2 > 0.978$ for molecular solution) (**Fig S2 B**).
