# Supplementary material for: Thermal Destabilization of Collagen Matrix Hierarchical Structure by Freeze/Thaw
Source: PLoS One. 2016 Jan 14;11(1):e0146660. doi: 10.1371/journal.pone.0146660 (PMC4713088; doi:10.1371/journal.pone.0146660)
Supplement: S3 Fig — (PDF) [file pone.0146660.s003.pdf]

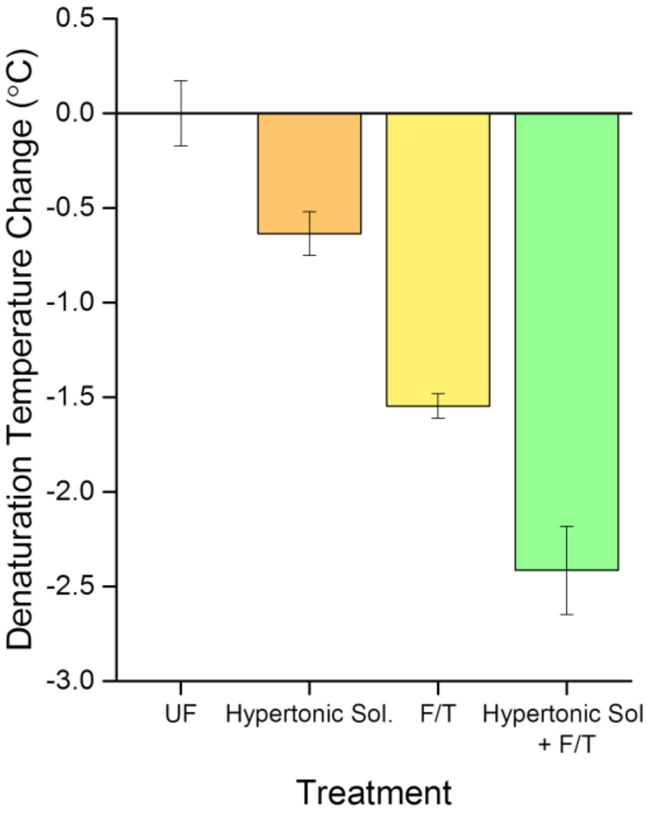

Fig S3. Denaturation temperature change with hypertonic solution treatment, F/T and combination of the two treatments.

Fig S3 shows the denaturation temperature change observed by hypertonic solution treatment, freeze/thaw (F/T) and the combination of the two treatments. A net decrease in denaturation temperature was observed with hypertonic solution treatment alone, even in the absence of F/T. However the magnitude of this change was only 0.5 °C, approximately one third of F/T effect. In addition, when hydrogels treated with hypertonic solution were also F/T, the magnitude of denaturation temperature decrease was found to be approximately the sum of the values for the individual hypertonic solution and F/T treatment. This suggests that the effects of the two treatments are additive and F/T and hypertonic treatments have independent mechanisms of action.
