# Supplementary material for: Thermal Destabilization of Collagen Matrix Hierarchical Structure by Freeze/Thaw
Source: PLoS One. 2016 Jan 14;11(1):e0146660. doi: 10.1371/journal.pone.0146660 (PMC4713088; doi:10.1371/journal.pone.0146660)
Supplement: S1 Table — (PDF) [file pone.0146660.s004.pdf]

Table S1. Collagen thermal denaturation peak characteristics for selected treatments. Values are reported as mean ± standard deviation.

Sample	Treatment	DMSO* Conc. (M)	T_d ** (°C)	$\Delta T_{1/2}$ (°C)	ΔH_d (J/g collagen)
Hydrogel	F/T -60°C	0	44.17 ± 0.07	3.00 ± 0.13	114 ± 23
	F/T -60°C	0.25	44.16 ± 0.35	3.17 ± 0.81	88 ± 43
	F/T -60°C	0.50	45.19 ± 0.16	2.92 ± 0.21	80 ± 16
	F/T -60°C	1.00	45.41 ± 0.16	2.46 ± 0.49	53 ± 45
	UF	-	45.71 ± 0.32	2.57 ± 0.23	79 ± 28
Molecular Solution	F/T -60°C	-	38.76 ± 0.07	1.97 ± 0.05	52 ± 2
	UF	-	38.59 ± 0.04	1.99 ± 0.05	48 ± 7

* Dimethyl sulfoxide (ME_2SO)

** T_d : denaturation temperature, $\Delta T_{1/2}$: peak width at half height, ΔH_d : denaturation enthalpy.

Denaturation temperature, T_d was determined based on the location of maximum specific heat after subtraction of a linear baseline from the original signal. Peak width at half height, $\Delta T_{1/2}$ which indicates the temperature span of the transition and denaturation enthalpy, ΔH_d i.e. the area under the endothermic peak are also calculated from the baseline subtracted thermogram.
