# Supplementary material for: Thermal Destabilization of Collagen Matrix Hierarchical Structure by Freeze/Thaw
Source: PLoS One. 2016 Jan 14;11(1):e0146660. doi: 10.1371/journal.pone.0146660 (PMC4713088; doi:10.1371/journal.pone.0146660)
Supplement: S2 Table — The best fitting values of model parameters, reaction order, n, activation energy, Ea, and the pre-exponential factor, A, are reported for selected F/T and UF cases. (PDF) [file pone.0146660.s005.pdf]

Table S2. Kinetic model parameters. The best fitting values of model parameters, reaction order, *n*, activation energy E_a, and the pre-exponential factor *A* are reported for selected F/T and UF cases.

Sample	Treatment	ln(*A*) (*A* in 1/s)	E_a (kJ/mol)	*n*	R^2
Hydrogel	F/T -60°C	307 ± 10	821 ± 25	1.55 ± 0.14	> 0.996
	UF Control	262 ± 19	705 ± 49	1.10 ± 0.07	> 0.997
Molecular Solution	F/T -60°C	446 ± 24	1167 ± 62	1.26 ± 0.04	> 0.994
	UF Control	445 ± 18	1164 ± 51	1.11 ± 0.14	>0.978
